# Internet-Based Screening for Dementia Risk

**DOI:** 10.1371/journal.pone.0057476

**Published:** 2013-02-21

**Authors:** Jason Brandt, Campbell Sullivan, Larry E. Burrell, Mark Rogerson, Allan Anderson

**Affiliations:** 1 Department of Psychiatry and Behavioral Sciences, The Johns Hopkins University School of Medicine, Baltimore, Maryland, United States of America; 2 Department of Neurology, The Johns Hopkins University School of Medicine, Baltimore, Maryland, United States of America; 3 The Copper Ridge Institute, Sykesville, Maryland, United States of America; 4 Independent Practice, Niskayuna, New York, United States of America; 5 Samuel and Alexia Bratton Memory Clinic, William Hill Manor, Easton, Maryland, United States of America; University Of São Paulo, Brazil

## Abstract

The Dementia Risk Assessment (DRA) is an online tool consisting of questions about known risk factors for dementia, a novel verbal memory test, and an informant report of cognitive decline. Its primary goal is to educate the public about dementia risk factors and encourage clinical evaluation where appropriate. In Study 1, more than 3,000 anonymous persons over age 50 completed the DRA about themselves; 1,000 people also completed proxy reports about another person. Advanced age, lower education, male sex, complaints of severe memory impairment, and histories of cerebrovascular disease, Parkinson's disease, and brain tumor all contributed significantly to poor memory performance. A high correlation was obtained between proxy-reported decline and actual memory test performance. In Study 2, 52 persons seeking first-time evaluation at dementia clinics completed the DRA prior to their visits. Their responses (and those of their proxy informants) were compared to the results of independent evaluation by geriatric neuropsychiatrists. The 30 patients found to meet criteria for probable Alzheimer's disease, vascular dementia, or frontotemporal dementia differed on the DRA from the 22 patients without dementia (most other neuropsychiatric conditions). Scoring below criterion on the DRA's memory test had moderately high predictive validity for clinically diagnosed dementia. Although additional studies of larger clinical samples are needed, the DRA holds promise for wide-scale screening for dementia risk.

## Introduction

Early detection of dementia is essential for early diagnosis and treatment, key interventions for managing disease and limiting morbidity [1], [2]. Recent years have seen calls for more widespread screening of at-risk populations (e.g., those over age 75 or those with family histories of dementia) [2], [3], [4].

Several dementia screening methods have been proposed [3], [5], [6], [7]. Some of these are intended to detect prevalent cases. They consist of cognitive tests [8], [9], [10], [11], [12] or informant questionnaires [6], [13], [14], [15] to efficiently screen large numbers of people for the presence of cognitive or functional decline. Persons detected by these procedures can then undergo more rigorous clinical examination [16], [17]. Other screening tests estimate one's risk for developing dementia in the future based on the presence or absence of established risk factors [18], [19], [20]. Many of the newest and most promising methods for detecting current or future dementia require invasive procedures (genetic and other biomarkers derived from serum or cerebrospinal fluid), expensive imaging, and/or lengthy neuropsychological assessments [21], [22]. However, a recent review [23] found existing methods to have limited predictive accuracy and called for the development of ‘parsimonious and cost effective consensus models.’

To answer this call, we developed the Dementia Risk Assessment (DRA). This very brief assessment of risk factors and cognitive performance does not require an in-person interview, physical examination, or biological samples, and could therefore be entirely automated and offered free of charge. Preliminary results in a small sample found advanced age, male sex, hypertension and complaint of severe memory impairment to be significant independent predictors of cognitive impairment [24]. The present report describes the performance of >3,000 anonymous older adults on our online DRA, as well as preliminary results from a validation study of persons seeking clinical evaluation for possible dementia.

## Methods

### Instrument Development

The DRA is an Internet-based assessment that collects three types of information to determine whether one is at increased risk of dementia: history of established health risk factors; a standardized, validated, informant report of cognitive decline; and a novel, very brief test of associative memory. The health risk factors consist of 12 neurological conditions, three medical disorders (hypertension, hypercholesterolemia, and diabetes) and two psychological disorders (depression and anxiety). These specific conditions (see Table 1) were selected based on the investigators' assessment of the factors most often shown to be strongly associated with cognitive impairment in previous studies [18], [19], [20], [23], as well as their own research interest in the cognitive effects of specific neurological disorders [25], [26], [27]. Participants answered the health and other risk questions as they pertain either to themselves (on the ‘patient page’) or to a relative/friend (on the ‘proxy page’). A set of decision rules was then applied, resulting in narrative feedback to participants about their various risk factors and qualitative statements about their overall risk of having or developing dementia.

**Table 1:** Comparison of older respondents from the Anonymous Internet Sample who were impaired (<10^th^ percentile) or unimpaired on the recognition memory test.

	Impaired	Unimpaired	*p* *	Odds Ratio†
N	309	2,859		
Age, mean (SD)	66.94 (10.46)	62.00 (8.29)	<.001	1.06 (1.05–1.07)
Sex (percent male)	46.93%	29.73%	<.001	2.09 (1.65–2.65)
Education, mean (SD)	14.75 (2.95)	15.74 (2.68)	<.001	0.87 (0.84–0.91)
Family history of dementia (% ‘yes’)	37.22%	40.78%	.432	
Severe memory problems (% ‘yes’)	23.95%%	8.53%	<.001	3.38 (2.52–4.52)
Personal history of (% ‘yes’)				
Neurological disorders:				
Stroke	5.50%	1.61%	<.001	3.56 (2.02–6.29)
Transient ischemic attack	8.41%	4.62%	.004	1.90 (1.23–2.94)
Traumatic brain injury	6.80%	4.93%	.158	
Epilepsy	1.62%	1.64%	.973	
Parkinson's disease	1.94%	0.74%	.028	2.68 (1.07–6.68)
Huntington's disease	0.32%	0.11%	.304	
Multiple sclerosis	0.00%	0.35%	.298	
Normal pressure hydrocephalus	6.47%	4.41%	.100	
Encephalitis	0.65%	0.74%	.864	
Brain tumor	1.94%	0.74%	.028	2.68 (1.07–6.68)
Any brain surgery	0.32%	0.94%	.268	
Any other brain disorder	0.97%	0.22%	.158	
Medical disorders:				
Hypertension	45.63%	38.79%	.019	1.32 (1.05–1.67)
Hypercholesterolemia	44.01%	41.76%	.446	
Diabetes	13.27%	10.25%	.101	
Psychological disorders:				
Depression	15.86%	16.61%	.734	
‘Nervousness’	30.10%	33.75%	.195	
Recognition Memory Test				
Score, mean (SD)	−0.03 (0.20)	0.72 (0.22)	<.001	
Delay Interval, mean min. (SD)	2.93 (1.81)	2.15 (1.07)	<.001	
IQCODE, mean (SD)	4.27 (0.61) (N = 16)	3.47 (0.55)(N = 30)	<.001	

* *p* values from t-tests (for continuous variables) or chi-square tests (for categorical variables).

† Univariate odds ratio (95% confidence interval).

Included on the patient page is a new, very brief, verbal memory test. The task requires the binding of objects with attributes, a cognitive mechanism that appears to depend on hippocampal functioning [28], [29] and may be particularly vulnerable in early Alzheimer's disease [30]. Participants read and attempt to memorize the names of six incongruently-colored objects (e.g., ‘pink mushroom’ and ‘blue lemon’). After 2–3 minutes of other interview questions, they are administered a yes/no recognition memory task. For each of the six target stimuli, there are two distracters. One is the pairing of the object with its usual color, presumably drawing on long-term semantic memory (e.g., ‘brown mushroom’ and ‘yellow lemon’). The other distracter is a different incongruent pairing (e.g., ‘yellow mushroom’ and ‘brown lemon’). Recognition accuracy is calculated as hit rate minus false-positive rate [31]. The resulting score can range from −1.0 to +1.0, with 0.0 being chance performance. Since the predictive value of memory test score was not yet established before this study was conducted, this score was not considered or included in the development of feedback statements.

Included on the proxy page is the 16-item version of the Informant Questionnaire on Cognitive Decline in the Elderly (IQCODE) [32], [33], a standardized and well-validated instrument for the detection of dementia in the community. Cut-off scores of 3.38 and higher were used to construct feedback statements that the person rated was likely displaying signs of cognitive impairment.

Twelve core feedback statements were developed, based on the subject's age (3 levels), complaint of severe memory loss (yes/no), and family history of dementia (yes/no). Each of these 12 feedback statements was supplemented with up to three additional paragraphs based on the presence or absence of one or more neurologic disorders, cerebrovascular risk factors (hypertension, hypercholesterolemia, or diabetes) or mental health disorder. Sample feedback statements appear in Appendix S1. Note that no specific numerical risk estimates are provided. Reports are generated for participants (both ‘patients’ and proxies) that contain recommendations, of varying strength, that patients seek formal evaluation of any significant cognitive complaints from their primary health care provider or a dementia specialist. The patient page and the proxy page each take approximately 5 minutes to complete.

### Study 1: Anonymous Internet Sample

The DRA was launched on the educational portal of the Copper Ridge Institute (www.alzcast.org/memorysurvey) in April 2009. Participants were informed that their anonymous responses were being collected for research purposes. Since no one's participation was solicited (i.e., people chose to visit the site and take the assessment on their own), and no personally identifying information was requested or collected, this aspect of the study did not require IRB approval. Data spreadsheets were downloaded from a secure, password-protected website on December 31, 2011. IRB approval was granted for analysis and publication of these anonymous data.

### Study 2: Clinical Validation Sample

Patients seeking first-time evaluation for possible cognitive disorder were recruited from two, private, not-for-profit, memory clinics located within retirement communities: Copper Ridge (in Sykesville, MD) and William Hill Manor (in Easton, MD). At each clinic, new patients were asked to complete the DRA at home prior to their first visit or, in some cases, at the time of the visit. They were then asked to bring a copy of the printed reports from the DRA to their clinic visit. At that visit, they gave permission for the research team to access their medical records and excerpt the results of their subsequent neuropsychiatric work up.

Clinical examinations were performed by experienced geriatric neuropsychiatrists. The exam consisted of obtaining a detailed history from the patient and collateral informants (typically the spouse and/or adult children); review of previous medical records; performing a physical exam, neurological exam, psychiatric interview, and mental status exam [including the Mini-Mental State Exam (MMSE)]; and obtaining necessary blood laboratory studies and brain imaging. Neuropsychiatric diagnoses were rendered using standard criteria and recorded using standard nomenclature [34], [35]. The DRA reports were not available to the clinicians performing the evaluations and making the diagnoses.

These procedures were fully reviewed and approved by Johns Hopkins University School of Medicine IRB, and all subjects and their legally authorized representatives (where appropriate) provided written informed consent.

## Results

### Study 1: Anonymous Internet Sample

**Figure 1:**
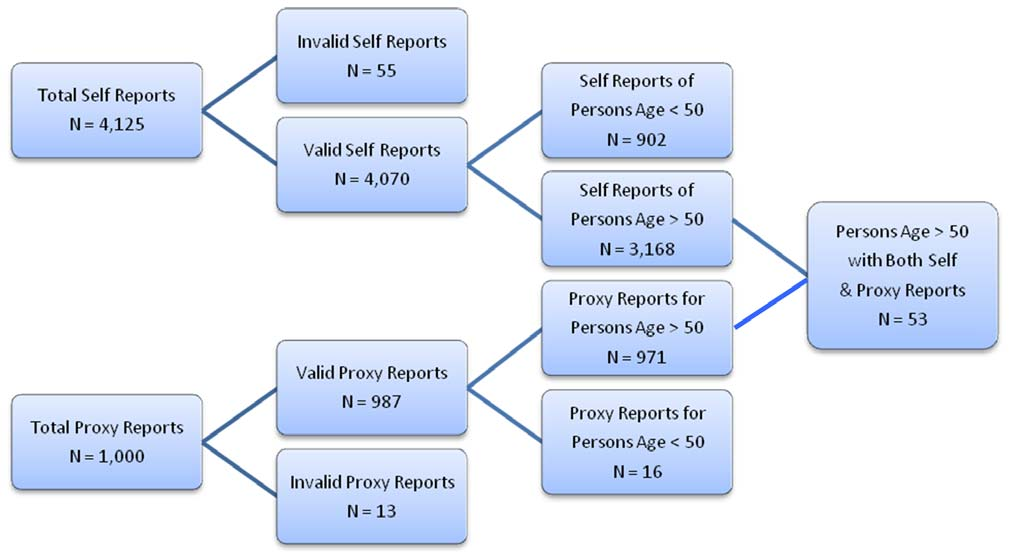
Anonymous Internet Sample.

A total of 4,125 self-report (‘patient’) pages were completed (see Figure 1). Fifty-five self-reports (1.3%) contained out-of-range or highly implausible responses (e.g., being age 102, having every neurological disorder queried), and their data were excluded. Respondents ranged in age from 18 to 97. The age distribution was essentially normal, with a mean of 57.2 years (SD = 13.2). The majority of respondents were women (68.1%), and they were generally well educated (mean highest grade completed  = 15.7, SD = 2.7). Approximately 11% of the sample reported having the equivalent of a doctoral degree (≥20 years of education).

Because the risk of cognitive decline is strongly associated with age, all further analyses were restricted to participants age 50 years or older (N = 3,168). Based on our pilot data [24], a score <0.25 on the recognition memory test was chosen as reflecting possible impairment, since it identified the lowest-performing 10% of the sample. A total of 309 subjects obtained scores within the impaired range. As a group, these subjects were significantly older, more likely to be male, less well educated, and more often had complaints of severe memory problems (see Table 1). They also more frequently reported histories of hypertension, stroke (CVA), transient ischemic attack (TIA), Parkinson's disease (PD), or brain tumor. A forward stepwise regression was performed on memory test score with these variables, plus memory test delay interval (time between last stimulus presentation and first yes/no test trial), as independent variables. A 7-variable linear model was statistically significant (F = 76.52, p<.0001), and accounted for 14.5% of the total variance in recognition memory score (see Table 2).

**Table 2:** Summary of stepwise multiple regression on recognition memory test score among participants age 50 and older in the Anonymous Internet Sample.

Step	Variable Entered	Response/Coding	β	R	R^2^ change	R^2^total
1	Age	Years	−0.181	0.260	0.068	0.068
2	Severe memory problems	‘Yes’	−0.132	0.307	0.026	0.094
3	Sex	Male	−0.158	0.335	0.018	0.112
4	Education	Years	0.117	0.358	0.016	0.128
5	Memory delay interval	Minutes	−0.121	0.376	0.013	0.141
6	Stroke	‘Yes’	−0.050	0.379	0.003	0.144
7	Parkinson's disease	‘Yes’	−0.033	0.381	0.001	0.145

One thousand proxy pages were completed by the Anonymous Internet Sample (Figure 1). A total of 971 of these were ostensibly valid reports on persons age 50 or older. The subjects of these proxy reports had a mean age of 75.3 (SD = 10.7), and a mean of 13.6 years of education. Their average score on the IQCODE was 3.88 (SD = 0.64). The significant predictors of IQCODE score in this sample were the respondent's report that the subject had severe memory problems (not surprising, since both reflect perception of cognitive decline), report that the subject seems ‘downhearted and sad’ ‘a good bit of the time’ or more, and reported history of TIA, diabetes, PD, epilepsy, and hypertension (F = 63.67, p<.0001). Together, these seven variables accounted for 32% of the total variance in IQCODE scores.

Fifty-three persons in the Anonymous Internet Sample completed a DRA self-report (on the patient page) and had a proxy page completed about them. [Persons taking the DRA for themselves and having one completed about them were asked to use the same ID number. Fifty-three patient-reports and proxy-reports used the same ID number. We have no way of knowing whether there are more ‘pairs’ in the database where the patient and proxy did not used the same ID number.] Their mean age was 73.7 (SD = 9.8), they completed an average of 14.4 years of education (SD = 3.0), their mean memory test score was 0.35 (SD = 0.38), and their mean IQCODE score was 3.73 (SD = 0.69). This very small sample precluded the development of multivariate predictive models. The bivariate correlation between IQCODE completed by the proxy and recognition memory test score was r = −.59 (p<.0001). This is considered a large statistical effect [36], and provides additional support for the validity of the DRA's recognition memory test.

### Study 2: Clinical Validation Sample

**Figure 2:**
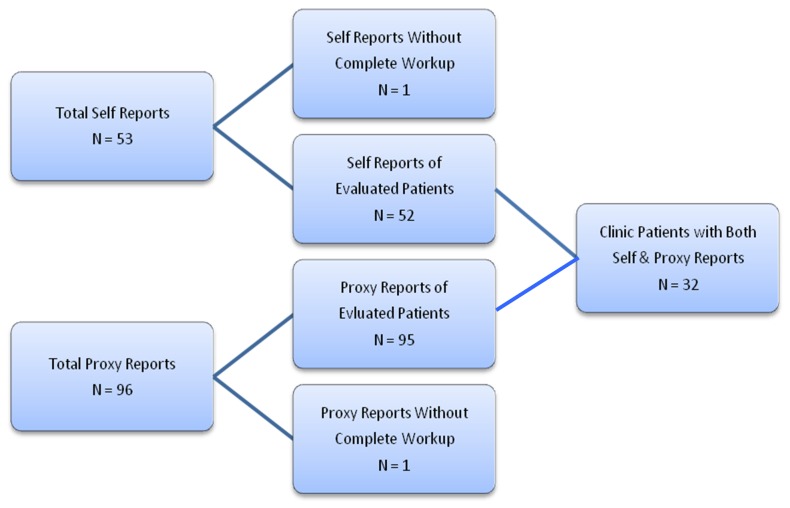
Clinical Validation Sample.

As of December 31, 2011, 52 new memory clinic outpatients who subsequently received state-of-the-art dementia evaluations completed the self-report of the DRA (see Figure 2). Their demographic and clinical characteristics are shown in Table 3. After these patients were fully assessed, 18 were diagnosed with probable AD and 12 were formulated as having a non-Alzheimer's dementia (vascular or frontotemporal dementia). Thirteen were formulated as having mild cognitive impairment (MCI), and two patients had other neurologic disorders (progressive supranuclear palsy and status post meningioma resection). Four patients were diagnosed with a primary psychiatric disorder (major depression in three and attention deficit disorder in one), and three patients did not meet criteria for any disorder.

**Table 3:** Characteristics of 52 outpatients in Clinical Validation Sample who completed the self-report (‘patient page’) of the Dementia Risk Assessment.

Age, mean (SD)	75.96 (9.47)
Sex (percent male)	36.53%
Education, mean (SD)	13.75 (3.03)
Family history of dementia (% ‘yes’)	30.77%
Severe memory problems (% ‘yes’)	38.46%
Personal history of (% ‘yes’)	
Neurological disorders:	
Stroke	15.38%
Transient ischemic attack	21.15%
Traumatic brain injury	5.77%
Epilepsy	1.92%
Parkinson's disease	1.92%
Huntington's disease	0%
Multiple sclerosis	0%
Normal pressure hydrocephalus	3.85%
Encephalitis	0%
Brain tumor	0%
Any brain surgery	0%
Any other brain disorder	3.85%
Medical disorders:	
Hypertension	55.77%
Hypercholesterolemia	40.38%
Diabetes	30.77%
Psychological disorders:	
Depression	21.15%
‘Nervousness’	23.08%
Recognition Memory Test	
Score, mean (SD)	0.27 (0.36)
Delay Interval, mean min. (SD)	3.46 (1.47)
IQCODE, mean (SD)	4.12 (0.67) (N = 32)

On the recognition memory test, the mean score for this sample was 0.22 (SD = 0.31). This is considerably lower than the average for 3,168 age-, sex-, and education-matched persons from the Anonymous Internet Sample (mean = 0.65, SD = 0.31). Memory test score was significantly correlated with both MMSE score (Pearson r = 0.51, p<.0001) and IQCODE (Pearson r = −0.49, p = .005) in this Clinical Validation Sample.

**Figure 3:**
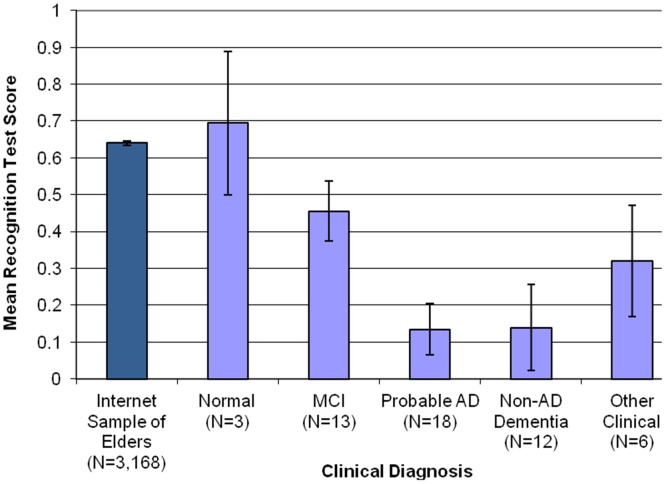
Recognition memory test scores (means ± standard errors) for the Clinical Validation Sample, as a function of subsequently-assigned clinical diagnosis. Performance of ≥50 year-old participants from the anonymous Internet sample shown for comparison.

When compared on the dementia risk factors queried, the 30 patients with dementia (AD or non-AD) differed from the 22 without dementia only in the prevalence of hypercholesterolemia (lower in those with dementia) and complaint of severe memory problems (higher in those with dementia) (data not shown). On the recognition memory test, the two dementia groups (probable AD and non-AD) were both severely impaired (see Figure 3). As expected, the MCI group had a mean performance intermediate between the demented and normal subjects. In this small sample, the sensitivity of the memory test to dementia (i.e., recognition memory <0.25) was 68% and its specificity was 63%. Using a higher cut-off (<.29) resulted in the same sensitivity but slightly better specificity (67%). The area under the receiver operating characteristic (ROC) curve was 0.75. In this sample, 68% of ‘positive’ tests (scores below 0.29) came from cases (persons diagnosed with dementia) (positive predictive value, PPV), while 67% of ‘negative’ tests came from noncases (persons with other conditions, including MCI) (negative predictive value, NPV).

## Discussion

The present report describes the utility of an entirely automated, very brief assessment of common dementia risk factors and memory performance in a sample of >3,000 older adults, and provides preliminary results from a validation study of persons seeking evaluation for possible dementia. We found that age, sex, education, complaints of severe memory problems, and reported histories of cerebrovascular disease (hypertension, stroke and transient ischemic attack), Parkinson's disease (PD), and brain tumor all differentiated normal from low memory performance. Although our predictive model accounts for only 14.5% of the total variance in memory score, this is considered a medium effect and is comparable to that obtained with in-person administration of traditional cognitive tests [37], [38].

There have been several previous attempts to develop algorithms for the prediction of dementia risk. Kivipelto and colleagues [20] tested regression models based on data collected in mid-life for dementia risk 20 years later. They found that age, education, apolipoprotein E (ApoE) status, systolic blood pressure, body mass index, total cholesterol, and level of physical activity could be combined to produce a total score that predicts up to a 16-fold increased risk of dementia. Barnes and colleagues [19] developed a late-life dementia risk index that stratifies older adults into low, moderate, or high risk of developing dementia within 6 years. Age, cognitive test performance, body mass index, ApoE status, findings of white matter disease or ventricular enlargement on brain MRI scans, carotid artery thickening on ultrasound, history of bypass surgery, slowed physical performance, and total abstinence from alcohol all contributed to the index score. Four percent of older adults with low scores on this index developed dementia within 6 years, compared with 23% of those with moderate scores, and 56% of those with high scores. Note that both of these models require DNA analysis and other biomedical assessments.

The results of our clinical validation study suggest that the DRA holds promise in the identification of cognitive disorders diagnosed by the ‘gold standard’: formal evaluation by a geriatric neuropsychiatrist, including supporting laboratory and imaging studies. DRA recognition memory test performance was highly correlated with both in-person cognitive performance (MMSE) and caregiver ratings of cognitive decline (IQCODE). The criterion validity of our memory test is established by its clear differentiation of persons seeking clinical evaluations who are found to meet criteria for AD or another dementia syndrome from those who do not (including persons with MCI and neuropsychiatric conditions). In our small dementia clinic sample, where the base-rate of cognitive disorder was high, a ‘positive’ test (i.e., scoring below .25) had 68% predictive validity for a dementia diagnosis.

The entirely automated and highly accessible format of the Dementia Risk Assessment may make it useful in large-scale screening programs, as might be required to identify at-risk elderly for inclusion in prevention trials. It also provides a way for those who are unable or unwilling to visit a dementia specialist to learn about their risk factors, and encourages those at high risk to seek clinical evaluation. The DRA may also provide reassurance to those concerned about developing dementia but whose empirical risk is low. It should be emphasized, however, that the DRA does not attempt to diagnose dementia, and this is clearly stated by the program.

Several limitations of the DRA instrument and of this study must be acknowledged. First, in an effort to keep the DRA brief (and thereby encourage its completion), several important risk-factors were excluded. Future versions might include additional health and lifestyle variables, such as body mass index, alcohol and tobacco use, physical exercise, and mentally stimulating activity. Second, although our Anonymous Internet Sample was open to persons anywhere in the world who are competent in English and who had access to the Internet, the representativeness of our two research samples and their comparability to future users is unknown. Third, there are limitations inherent in the online administration. Although Internet-based assessments are becoming increasingly prevalent and results demonstrate good validity [39], the conditions of administration are not controlled and there is no way to ensure legitimate results. It is certainly feasible that some participants did not tell the truth on risk factor questions or violated instructions to obtain higher scores on the memory test. Alternatively, poor performances may have been due to environmental distracters or waning motivation, rather than genuine memory impairment. Other individuals may have completed the DRA on more than one occasion. Given that participants are self-selected, it may be argued that the vast majority of respondents who are sufficiently motivated to engage in online dementia screening would complete the instrument honestly.

Probably the most useful data on dementia risk are those provided by self- and proxy-reports about the same person, and comparing self-report ratings with informant reports will be important for future studies. The number of such pairs in our existing Internet sample is extremely small, probably due in part our desire to keep the data anonymous. Overcoming this limitation will be challenging, but the continued evolution of technology may allow us to address this issue in future versions of the DRA. Finally, the size in our Clinical Validation Sample was small and did not allow us to test robust predictive models. A larger-scale clinical validation study is currently underway.

## Supporting Information

Appendix S1 **Sample feedback statements from Dementia Risk Assessment.**
(DOCX)
