# Supplementary material for: Internet-Based Screening for Dementia Risk
Source: PLoS One. 2013 Feb 21;8(2):e57476. doi: 10.1371/journal.pone.0057476 (PMC3578821; doi:10.1371/journal.pone.0057476)
Supplement: Appendix S1 — Sample feedback statements from Dementia Risk Assessment. (DOCX) [file pone.0057476.s001.docx]

**APPENDIX**

Sample Feedback Statements

Case 1: Patient Page (Self-Report), 60 year-old woman, no family history of dementia, complaints of severe memory problem, acknowledged depression and anxiety

“Alzheimer’s disease (AD) is primarily a disease of older people. Less than 1% of the population under age 65 has AD. If you are in this age range and have severe memory difficulties, a disorder other than AD may be more likely. Consider any of the risk factors described below. Between ages 65 and 74, the prevalence of AD (proportion of the population with the disorder) increases to approximately 2%. Since you report having severe memory problems, you are strongly encouraged to seek formal evaluation by a dementia specialist. You can probably get a recommendation from your primary health care provider.

Any difficulties you may be having with memory or other cognitive abilities may be the result of emotional factors. Research has indicated that persons with depression, anxiety, or other types of psychological distress often have trouble concentrating and remembering. Moreover, these psychological states often “color” our self-perceptions. For example, people who are depressed often see themselves in a negative light and rate their cognitive abilities as worse than they actually are. Consider your current life situation. Have you experienced a recent loss or another type of emotionally traumatic event? Have you received mental health counseling or psychological/psychiatric treatment in the past? You might want to discuss your memory problems with your physician, or seek the assistance of a mental health professional.”

Case 2: Patient Page (Self-Report), 80 year-old man, hypertension and hypercholesterolemia, family history of dementia, no complaints of severe memory problems or mental health problems

“Between the ages of 75 and 84, the prevalence of Alzheimer’s disease (AD) (proportion of the population affected) is approximately 20%. Over age 85 and older, this increases to approximately 42%. This illustrates the profound effect that aging has on the prevalence of AD.

You indicated that you do not have a severe memory problem. That’s good to hear! However, 3 out of 4 people *with* cognitive impairment or dementia also say they don’t have severe memory problems. That’s because many people with dementia, even in the earliest stages, are not fully aware of their difficulties. So while your response is *encouraging*, it does not allow us to rule out the possibility that you have a cognitive disorder. The fact that you have one or more first-degree relative with dementia makes the chance that you will develop dementia in the future a bit higher than if you did not have a relative with dementia.

High blood pressure, high cholesterol, and diabetes are all significant risk factors for cerebrovascular disease, a condition where blood supply to the brain is inadequate. After Alzheimer’s disease, cerebrovascular disease is the most common cause of cognitive impairment in older adults. This type of dementia goes by various names, including “vascular dementia” and “multi-infarct dementia.” There is also increasing evidence that cerebrovascular disease contributes to the development of AD. Since you have one or more of these conditions, your risk for the development of dementia is increased; exactly how high it is cannot be determined. However, there are things you can do to minimize your risk. Take any medications you are prescribed for these conditions exactly as prescribed. Follow your doctor’s recommendations regarding diet and exercise. In general, health habits that are good for your heart are also good for your brain.”

Case 3: Proxy Page (Rater-Report), 50 year-old woman with seizure disorder, no family history of cognitive impairment, IQCODE >3.38 (i.e., impaired)

“Alzheimer’s disease (AD) is primarily a disease of older people. The likelihood that the subject is showing signs of AD is extremely low, especially since she apparently has no family history of dementia. Does the subject have other physical health problems that might be affecting her cognitive functioning? Consider also any medications that she may be taking; ask her doctor whether any of them may be contributing to mild or moderate problems with thinking or memory problems. Does the subject have any problems with substance abuse? Excessive use of alcohol or other drugs that cause intoxication certainly interfere with brain functioning and can have lasting effects (even when the subject is sober).

You indicated that the subject has a history of epilepsy. It is well established that epilepsy or the medications used to treat it, can be associated with cognitive difficulties. Discuss the subject’s cognitive symptoms with her neurologist or other physician who manages her care.”
